# Unique case of metastatic synovial sarcoma to the breast: Case report and review of the literature

**DOI:** 10.1016/j.ijscr.2025.111567

**Published:** 2025-06-24

**Authors:** Lara AlKhelaiwy, Faris AlSalamah, Abdulmohsen AlKushi, Khalid AlHajri

**Affiliations:** a General Surgery, Specialized Medical Center, Riyadh, Saudi Arabia; b King Abdullah International Medical Research Center (KAIMRC), Riyadh, Saudi Arabia; c College of Medicine, King Saud Bin Abdulaziz University for Health Sciences, Riyadh, Saudi Arabia; d Department of Pathology and Laboratory Medicine, King Abdulaziz Medical City and King Saud bin Abdulaziz University for Health Sciences, Riyadh, Saudi Arabia; e General Surgery/Breast and Endocrine, Prince Sultan Military Medical City, Riyadh, Saudi Arabia

**Keywords:** Synovial sarcoma, Metastasis, Breast, Nonhematological, Extramammary, Neoplasms metastasis to breast, Nonmammary metastases

## Abstract

**Introduction and importance:**

Synovial sarcoma is a rare and aggressive soft tissue cancer primarily affecting young adults, and metastasis to the breast from a primary tumor in the extremities is exceedingly rare. We report a rare case of right knee synovial sarcoma with metastasis to the left breast occurring five years after initial diagnosis.

This work has been reported in line with the SCARE 2023 criteria [20].

**Case presentation:**

We present the case of a 36-year-old female who developed metastatic left breast sarcoma five years after her initial diagnosis of right knee synovial sarcoma, which was treated with wide local excision. She underwent a left partial mastectomy. Her postoperative recovery was uneventful. A PET-CT scan performed during her 16-month follow-up period showed no evidence of metastasis or recurrence.

**Clinical discussion:**

This case describes a rare instance of synovial sarcoma metastasizing to the breast, highlighting the importance of including metastatic disease in the differential diagnosis of breast masses, especially in patients with a history of malignancy. The case was initially misdiagnosed as epithelioid leiomyoma, underscoring the diagnostic challenge due to overlapping features.

Diagnosis was confirmed through cytological comparison with the primary knee tumor and immunohistochemistry. With only seven similar cases reported in the literature, there are no established guidelines for diagnosis or treatment of metastasis breast sarcoma.

**Conclusion:**

Due to the lack of standardized guidelines for managing breast metastases of synovial sarcoma, a multidisciplinary approach incorporating systemic therapy, surgery, and close surveillance is essential for personalized treatment.

## Highlights

• Breast metastasis from an extramammary primary malignancy is very rare.
• Synovial sarcoma metastasis to the breast is extremely rare with only 7 cases reported.
• Rare breast metastases should be considered, especially if there is a history of other primary cancer.
• Careful evaluation of clinical, imaging, and histopathological characteristics is essential for an accurate diagnosis.

## 1 Introduction

Synovial sarcoma is an aggressive soft tissue sarcoma primarily affecting adolescents and young adults [1]. It accounts for 5 %–10 % of soft-tissue sarcomas and most commonly arises in the extremities, with less frequent sites including the head, neck, and peritoneum [2]. Despite local control with surgery and radiotherapy, synovial sarcoma carries a high risk of metastasis and is generally considered a high-grade sarcoma marked by a poor prognosis [3]. In 50 % of cases, distant metastasis manifests either at the time of diagnosis or within few years [4]. The lungs are the most common site of metastasis, followed by lymph nodes, bone, and rarely, the liver and brain [4,5].

While primary breast tumors exhibit diverse and well-documented cytological features, secondary malignancies in the breast are less common and not as well understood [6]. Metastasis to the breast from extramammary non-hematological tumors is rare and associated with a poor prognosis. These metastases mainly originate from carcinomas, followed by melanomas and sarcomas [7]. Notably, breast metastasis from synovial sarcoma is exceedingly rare.

Here, we present a unique case of synovial sarcoma of the knee with metastasis to the breast, occurring five years after the initial diagnosis.

## 2 Case presentation

**Fig. 1:**
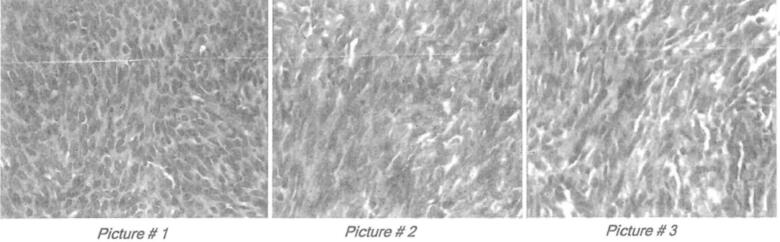
Histopathology of right knee tumor. Gross picture: grayish white firm mass, around 5.8 × 2 cm. Microscopic examination: tumor mass formed of spindle shaped cells, and round to polygonal cells arranged in pseudo epithelioid cords between bands of hyalinized stroma. There are sheets of cells with clear cytoplasm, there is no malignancy.

A 36-year-old female with a medical history of hypothyroidism and no history of trauma, presented five years ago with a two-month history of progressively worsening right knee pain, particularly with knee extension, along with increasing swelling in the area. On examination, a 4 cm round, non-tender, mobile soft mass was identified on the medial side of the right knee. Magnetic Resonance Imaging (MRI) revealed a prepatellar mass measuring 4 × 3.5 × 2 cm. After excision of the mass at a different facility, histopathological analysis initially suggested epithelioid leiomyoma (Fig. 1), though no immunohistochemical testing was performed at that time.

Approximately one year after the surgery and initial diagnosis of right knee epithelioid leiomyoma, the patient presented to our institution with a recurrent mass at the same site. Re-evaluation of the original histopathology slides, with the addition of immunohistochemical staining, revealed findings consistent with synovial sarcoma. The tumor was characterized by spindle cells with amphophilic cytoplasm, oval nuclei with prominent mitotic activity, and foci of tumor necrosis. Immunohistochemistry showed positive TLE1, EMA, CD99, and SOX10 staining, while staining for S100, desmin, cytokeratin (CK), CD34, and HMB45 were negative. These immunohistochemical findings are characteristic of synovial sarcoma, confirming the diagnosis.

MRI and PET-CT scans demonstrated local recurrence involving the right quadriceps and patellar tendons, with no evidence of distant metastasis. The patient subsequently underwent radiation therapy followed by wide-margin resection of the right knee tumor and reconstruction with an allograft. The resected tumor was confirmed to be a grade III, 4 × 2 cm monophasic synovial sarcoma with clear margins.

One year after she first presented to our hospital and underwent her second wide local excision, a follow-up PET-CT and MRI scans revealed metastatic spread to the upper third of the right tibia and a left pulmonary nodule. Molecular profiling did not reveal any targetable mutations.

In response to disease progression, systemic chemotherapy was initiated with doxorubicin (20 mg/m^2^/day) for three days and ifosfamide (1500 mg/m^2^) for four days. After three cycles, however, the disease continued to progress, leading to a switch to second-line therapy with eribulin (1.23 mg/m^2^) on days 1 and 8 every three weeks. After eleven cycles of eribulin, PET-CT scans showed disease stabilization, leading to a temporary cessation of treatment with plans for reassessment in three months. Unfortunately, follow-up scans three months later revealed metabolic progression of both the left pulmonary and right tibial lesions. A biopsy of the tibial mass confirmed recurrent synovial sarcoma. As a result, a wide resection of the right tibial tumor was performed, along with stereotactic body radiotherapy (SBRT) for the lung nodules. Further treatment was then deferred due to the patient's pregnancy.

**Fig. 2:**
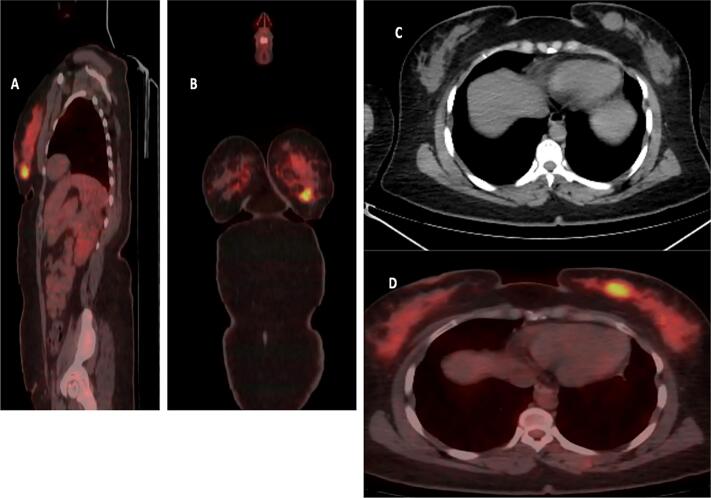
PET-CT scan sagittal (A), coronal (B), and axial (D) images shows a focal intense hypermetabolism in the left breast with standardized uptake value (SUV) max 8 corresponding to a 2.2 × 2 cm nodule in the lower outer quadrant, as seen on computed tomography (CT) scan (C).

**Fig. 3:**
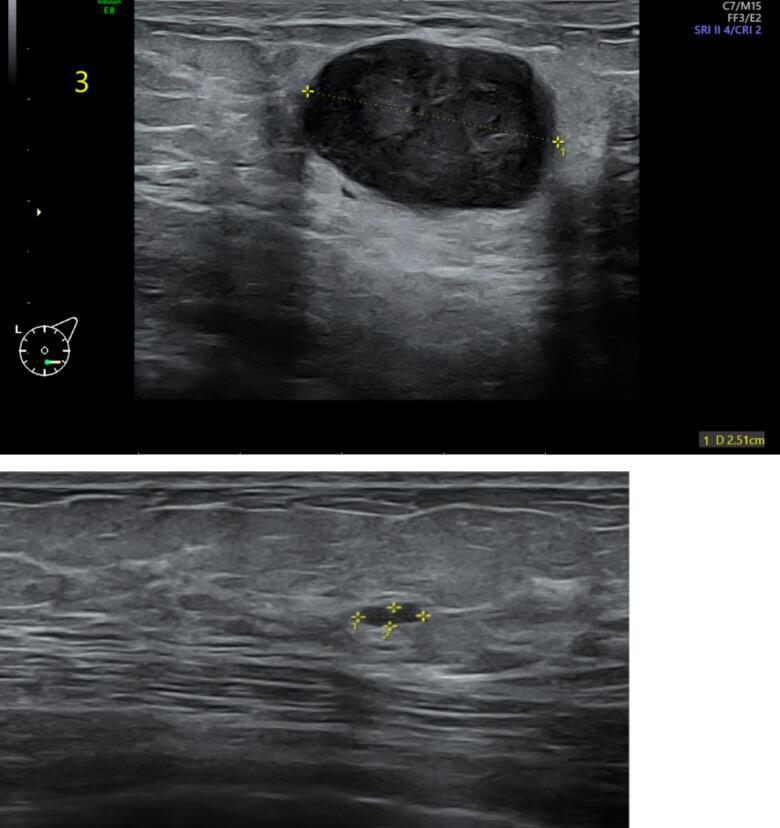
Ultrasound of the left breast showing breast mass at the 6:00 O'clock position, located 3 cm away from the nipple, 2.6 × 2.1 × 2.4 cm in size with heterogenous echotexture and posterior acoustic enhancement meriting a BI-RADS 4 classification.

**Fig. 4:**
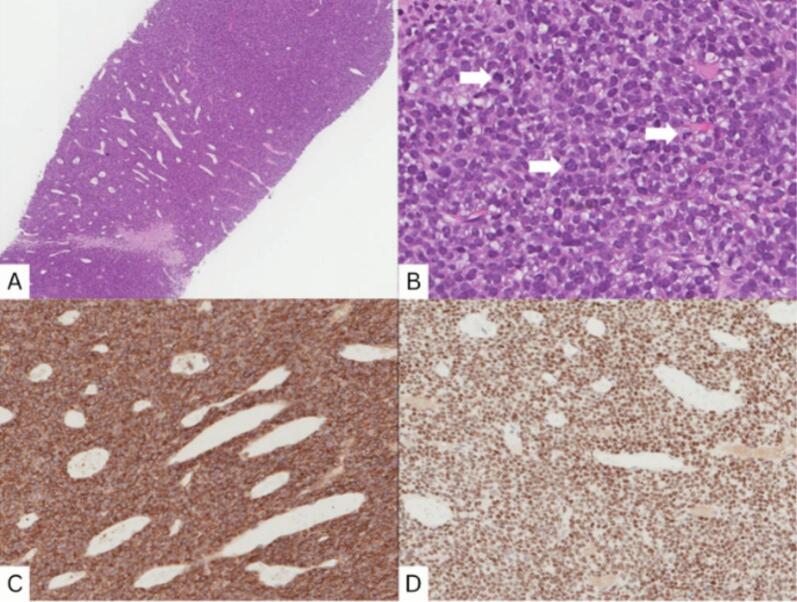
Histopathology and immunohistochemistry. Histopathology (H and E) showed lesional cells arranged in hypercellular sheets with prominent vasculature of thin-walled vessels with branching and stag-horn appearance (A). Tumor cells and monotonous, round to oval with moderate basophilic to clear cytoplasm, and the nucleus is hyperchromatic, round to oval with smooth nuclear contour with abundant mitosis (White arrows in B). Immunohistochemistry showed tumor cells positive for CD99 (C), TLE-1 (D).

After delivery, the patient developed a painless, approximately 2 cm mass in the left lower outer quadrant of her breast. Routine surveillance PET-CT imaging revealed a new hypermetabolic lesion in the left breast (Fig. 2), along with three right-sided pulmonary nodules. Notably, there was no fluorodeoxyglucose (FDG) uptake in the axillary lymph nodes, suggesting no nodal involvement. The previously treated left pulmonary nodule remained inactive. A subsequent breast ultrasound characterized the mass as a 2.6 × 2.1 × 2.46 cm lesion, warranting a BI-RADS 4 classification (Fig. 3). Core needle biopsy confirmed the diagnosis of metastatic synovial sarcoma, with the immunohistochemical profile mirroring that of the original knee tumor (Fig. 4).

**Fig. 5:**
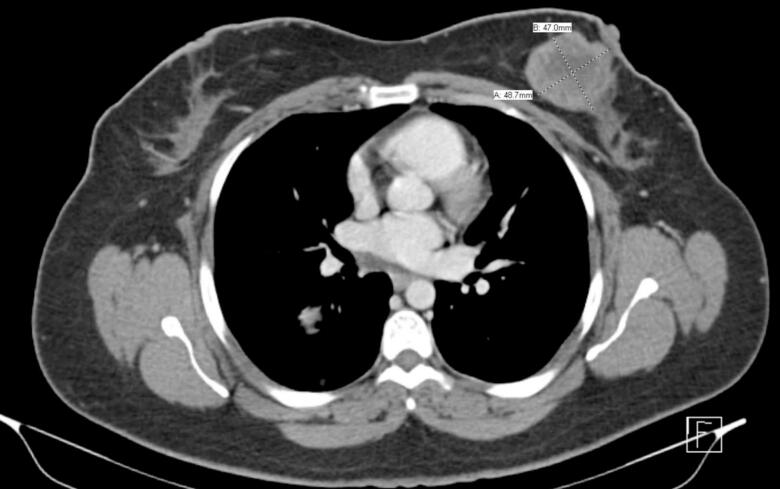
Progressive course of the previously seen soft tissue mass lesion of the left breast, its measuring 4.9 × 4.7 cm comparing to 3 × 3 cm previously, it shows moderate heterogenous enhancement.

After confirming metastatic synovial sarcoma by biopsy, eribulin therapy was resumed for 2 cycles. However, a follow-up chest CT scan showed an increase in the size of the left breast mass, while the right lung nodules remained stable (Fig. 5). Due to the progression of the breast lesion, the chemotherapy regimen was switched to third-line treatment with gemcitabine (675 mg/m^2^), docetaxel (75 mg/m^2^) and Tamoxifen (20 mg daily) was also added to the treatment regimen.

**Fig. 6:**
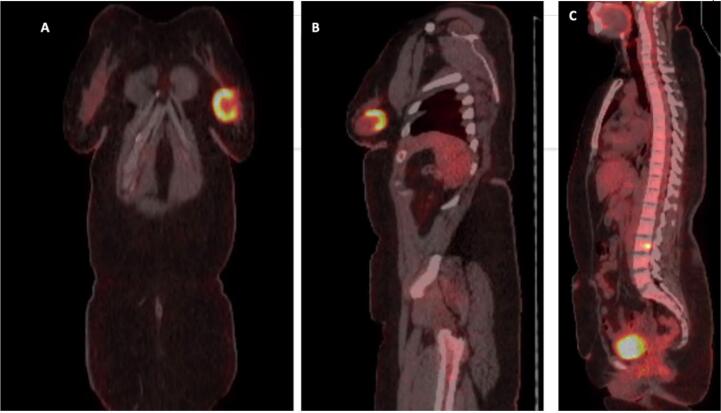
PET-CT scan coronal (A), and sagittal (B, C) showing interval increased size and uptake of the previously identified left breast mass lesion, measuring 4.8 cm compared to 2.2 cm with SUV max 10 compared to 8 previously, consistent with disease progression. And (C) showing newly apparent focal intense metabolically active bony lesion in the pedicle of L-3 lumbar vertebra with SUV max 6.5, probably bone metastasis.

Despite these interventions, a repeated PET-CT scan after chemotherapy demonstrated further disease progression. A new hypermetabolic lesion was detected at the left pedicle of the L3 vertebra, with an increasing size and metabolic activity of the left breast mass. At this point, the size was 4.8 cm with an SUVmax of 10 (Fig. 6). However, there was concurrent metabolic regression in the right pulmonary nodules.

Given the disease progression despite three lines of chemotherapy, the decision was made to do metastasectomy for the residual pulmonary and breast lesions. This was accompanied by initiating pazopanib (800 mg daily) as fourth-line systemic therapy. The patient underwent wide local excision of the left breast mass (partial mastectomy), right thoracotomy with metastasectomy of all three pulmonary nodules, and excision of lymph nodes from stations 7 (right upper hilar) and 9 (right lower hilar). Histopathological evaluation confirmed synovial sarcoma in all resected specimens, with clear surgical margins and negative right thoracic lymph nodes.

**Fig. 7:**
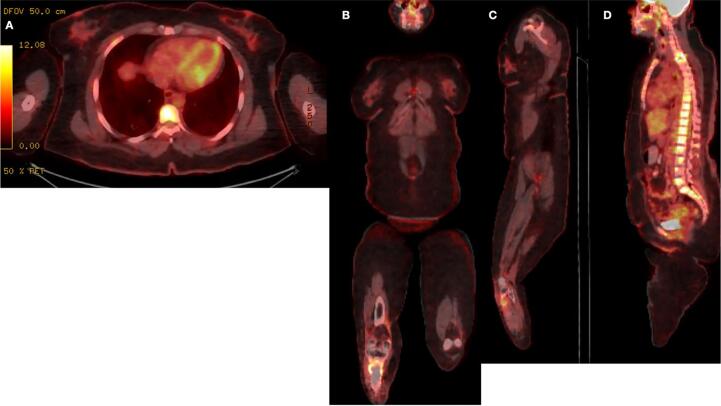
PET-CT scan (A, B, and C) showing no metastasis. Metabolic regression of the hypermetabolic bony lesion involving L-3 lumbar vertebra (D), stable diffuse mild activity in the right upper tibia along the bone graft with metallic fixation suggesting post-operative/reactive inflammatory changes (B, and C).

**Fig. 8:**
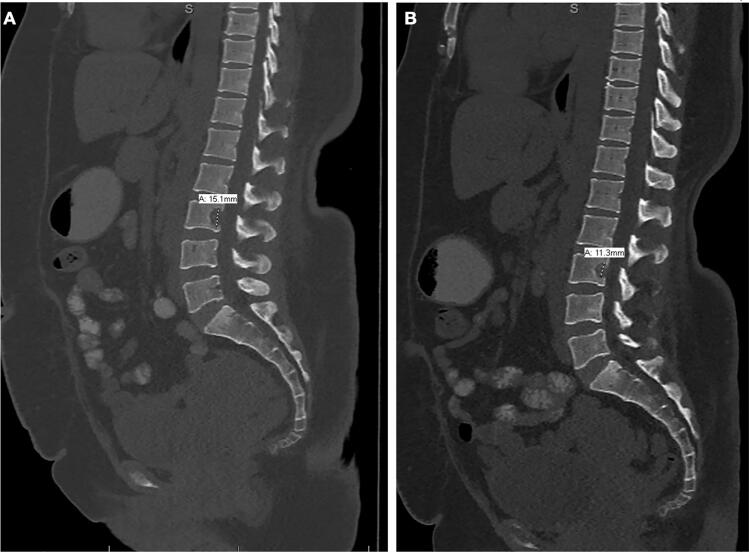
CT scan showing a slight increase in the size of the L3 lesion, measuring 1.5 cm (A), compared to 1.1 cm previously (B).

Although the most recent PET-CT scan (Fig. 7) showed metabolic regression in the L3 vertebral lesion and no new metastatic activity, the patient reported persistent back pain that was unresponsive to regular analgesics. Additionally, the latest CT scan (Fig. 8) revealed a slight increase in the size of the L3 lesion, measuring 1.5 cm compared to 1.1 cm previously. The patient subsequently underwent palliative radiation therapy to the L3 lesion, which she completed while continuing pazopanib. Following treatment, her pain has improved and is now responsive to regular analgesics.

Table 1 provides a comprehensive summary of the metastatic pattern of synovial sarcoma in this case, along with details of the patient's treatment and management.

**Table 1:** Summary of the metastatic pattern of synovial sarcoma in this case, along with details of the patient's treatment and management.

Site	Date	Management
Right knee	2018	Initial surgery with misdiagnosis of epithelioid leiomyoma
Right knee	2019	Revised diagnosis of synovial sarcoma, radiation therapy and wide-margin resection
Right tibial bone	2020	Systemic chemotherapy (Doxorubicin and Ifosfamide) 3 cycles, eribulin 11 cycles, and right tibial tumor resection
Left pulmonary nodule	2020	Systemic chemotherapy (Doxorubicin and Ifosfamide) 3 cycles, eribulin 11 cycles, and stereotactic radiosurgery
Left breast tumor	2023	2 cycles Eribulin, 2 cycles Gemcitabine/Taxotere, Tamoxifin 20 mg, Pazopanib 800 mg daily, partial mastectomy.
3 Right lung nodules	2023	2 cycles Eribulin, 2 cycles Gemcitabine/Taxotere, Tamoxifin 20 mg, Pazopanib 800 mg daily, thoracotomy and metastectomy of the 3 right lung tumors.
L-3 lumbar vertebra lesion	2024-prsent	Pazopanib 800 mg daily. Completed palliative radiotherapy.

## 3 Discussion

The case presented herein details the unusual occurrence of synovial sarcoma metastasizing to the breast, a rare site for secondary tumors from this soft tissue sarcoma. This case highlights the importance of maintaining a broad differential diagnosis for breast masses, particularly in patients with a history of malignancy. To reach a definitive diagnosis, we analyzed the lesion's cytomorphologic features, clinical data, and the primary tumor's previous cytological diagnosis and histopathology slides. To determine metastatic extramammary synovial sarcoma, we had to exclude primary breast soft tissue sarcomas. In the present case, examining the previous right knee tumor exhibited similar morphological traits and was sufficient to favor metastatic synovial sarcoma. However, a precise definitive diagnosis of synovial sarcoma was made using ancillary techniques; CD99 and TLE-1 could be detected immunohistochemically to confirm the diagnosis.

It is critical to differentiate between epithelioid leiomyoma and synovial sarcoma, as both can present with overlapping histological features. Epithelioid leiomyoma is a benign smooth muscle tumor characterized by uniform spindle cells with eosinophilic cytoplasm and positivity for smooth muscle markers such as desmin and smooth muscle actin (SMA). In contrast, synovial sarcoma exhibits a biphasic or monophasic spindle cell morphology with specific markers like TLE1, CD99, and Bcl-2, which aid in differentiation. The presence of SYT-SSX gene fusion further confirms synovial sarcoma, distinguishing it from other mesenchymal neoplasms [8]. In our case, the initial misdiagnosis as epithelioid leiomyoma highlights the importance of considering synovial sarcoma in the differential diagnosis of spindle cell lesions, especially in young patients.

The present case is extremely rare. After an extensive literature review, only seven cases of metastatic synovial carcinoma to the breast were identified (Table 2). Summarize the published cases of metastatic synovial sarcoma to the breast and compare them to the present case. Of all published cases, one patient was male [9], and the other six were female [[10], [11], [12], [13], [14], [15]]. Patients' ages at the time of breast metastasis diagnosis ranged from 58 to 24 years old, with a mean age of 33.7 years. On average, the size of the breast mass was 2.4 cm, and the time elapsed from the diagnosis of primary synovial sarcoma to the diagnosis of metastasis to the breast was 21.3 months, with one case of breast mass presenting simultaneously with the primary tumor and was the first manifestation of both malignancies [12]. The prognosis was difficult to compare as the disease outcome was only reported in three cases. The first case reported that the patient was well at six months follow-up [14], the second remained stable in size at follow-up after two years from completing 6 cycles of chemotherapy [15], while the other indicated that the patient died within two years of synovial sarcoma metastasis to the breast [12].

**Table 2:** Published cases of metastatic synovial sarcoma to the breast [[8], [9], [10], [11], [12], [13], [14]].

Case	Age/sex (*years*/*male*-*female*)	Clinical details	Dissemination	Location mammography	Primary tumor location	Time from the primary tumor	Management/outcome
Upadhyay et al., 2020 [10]	26/F	Found via follow-up PET/CT	No	Upper inner quadrant; left breast	Right lung; lower lobe.	6 months	Not reported
Ubayawansa et al., 2015 [11]	37/F	A clinically benign, well-circumscribed lump measuring about 2 cm.	No	Upper outer quadrant; right breast.	Right thigh	1 year	Not reported
Rodrígue z-Gil et al., 2012 [12]	58/F	A Painful 3-cm breast lump was the first manifestation of malignancy of the primary tumor.	Lung	Upper inner quadrant; left breast; well demarcated margins	Right leg	Simultaneously	Not reported/death within 2 years
Kijima et al., 2007 [13]	27/F	A 2 cm breast mass	Lung, after 28 months of primary	Upper outer quadrant; left breast.	Left knee	14 months	Excision then simple mastectomy due to recurrence/knee and breast recurrence 8 months after excision of 1st breast mets
Nair and Basu, 2005 [9]	24/M	A hitherto unknown 2.5-cm mobile nodule in the right breast.	No	Right breast	Right upper extremity	Not reported	Not reported
Banerjee et al., 2004 [14]	28/F	A 2.5 cm palpable lump	No	Upper outer quadrant; left breast. Well-defined solid mass with increased blood flow	Left lung; upper lobe	4 years	Total mastectomy/well at 6 months follow up
Khanal et al., 2024 [15]	42/F	Found via follow-up PET/CT	Recurrence to the left thigh, metastasis to the lungs bilaterally	Upper inner quadrant of the left breast	Left thigh	4 years	Ifosfamide, adriamycin, and cisplatin regimen/pulmonary nodules have decreased in size while left breast nodules are stable as of now.
Present case	36/F	2.6 cm breast mass	Lung, after 24 months of primary	Right upper outer quadrant.	Right knee	5 years	Chemotherapy, partial mastectomy/no recurrence as of now with slight increase in size of the L3 vertebral lesion

There are no established guidelines for the diagnosis and management of synovial sarcoma metastasizing to the breast due to its extreme rarity. In most reported cases, diagnosis is confirmed via histopathology and immunohistochemical staining, with markers such as Bcl-2, CD99, and TLE1 aiding in differentiation from primary breast malignancies [16]. Molecular confirmation using the SYT-SSX fusion gene is often necessary to definitively establish the diagnosis [17]. Imaging modalities such as PET-CT and MRI can assist in detecting occult metastases, but routine screening for breast involvement is not currently recommended in synovial sarcoma follow-up protocols [18].

Treatment strategies for breast metastases of synovial sarcoma remain controversial [13] [14] [15]. Unlike primary breast cancer, surgical excision with clear margins may not always be the preferred approach, as systemic disease control is often the primary goal [15]. Chemotherapy regimens remain the cornerstone of treatment for metastatic synovial sarcoma, though their efficacy in cases with breast involvement is not well defined [13] [15]. Radiotherapy may be considered in select cases, especially for local symptom control [19]. Given the lack of standardized treatment protocols, a multidisciplinary approach is essential to tailor therapy based on the patient's overall disease burden and clinical status.

## 4 Conclusion

Due to the lack of standardized guidelines for managing breast metastases of synovial sarcoma, a multidisciplinary approach incorporating systemic therapy, surgery, and close surveillance is essential for personalized treatment. This rare disease entity requires further investigation through case studies and clinical research to optimize diagnostic and therapeutic strategies. Physicians should maintain a high index of suspicion for this unusual breast neoplasm, especially in patients with a history of sarcoma, as it can mimic primary breast cancer. Additional case reports are crucial to enhance our understanding of this unique presentation, its response to treatment, and its prognosis.

## Author contribution

**Lara AlKhelaiwy**: writing the paper, data collection, data analysis, data interpretation.

**Faris AlSalamah**: writing the paper, data analysis, data interpretation.

**Abdulmohsen AlKhushi**, **MD**: study concept, investigation, resources, supervisor.

**Khalid AlHajri**: study concept, treatment of patient, editing the paper, supervisor.

## Consent

Written informed consent was obtained from the patient for publication of this case report and accompanying images. Patient confidentiality was strictly maintained throughout the study. A copy of the written consent is available for review by the Editor-in-Chief of this journal on request.

## Ethical approval

Ethical approval was waived by the institution's Research Ethics Committee. Case reports involving a single patient are exempt from ethics approval at our institution.

## Guarantor

Lara AlKhelaiwy.

## Sources of funding

This research did not receive any specific grant from funding agencies in the public, commercial, or not-for-profit sectors.

## Research registration

None.

## Declaration of competing interest

The authors declare that there is no conflict of interest.
